# Experimental proof of emergent subharmonic attenuation zones in a nonlinear locally resonant metamaterial

**DOI:** 10.1038/s41598-020-68894-3

**Published:** 2020-07-21

**Authors:** Valentina Zega, Priscilla B. Silva, Marc G. D. Geers, Varvara G. Kouznetsova

**Affiliations:** Eindhoven University of Technology, P.O. Box 513, 5600 MB Eindhoven, The Netherlands

**Keywords:** Applied physics, Materials science

## Abstract

High-performance locally resonant metamaterials represent the next frontier in materials technology due to their extraordinary properties obtained through materials design, enabling a variety of potential applications. The most exceptional feature of locally resonant metamaterials is the subwavelength size of their unit cells, which allows to overcome the limits in wave focusing, imaging and sound/vibration isolation. To respond to the fast evolution of these artificial materials and the increasing need for advanced and exceptional properties, the emergence of a new mechanism for wave mitigation and control consisting in a nonlinear interaction between propagating and evanescent waves has recently been theoretically demonstrated. Here, we present the experimental proof of this phenomenon: the appearance of a subharmonic transmission attenuation zone due to energy exchange induced by autoparametric resonance. These results pave the path to a new generation of nonlinear locally resonant metamaterials.

## Introduction

Metamaterials^1, 2^ are artificially engineered materials designed to obtain specific, often exotic, properties. Among the wide variety of possible metamaterial properties, the opening of a band gap, which is defined as the frequency range where elasto-acoustic waves cannot propagate, is attracting increasing interest because of its versatile applications^3–5^. In particular, a wide and low frequency band gap offers a number of potential applications, such as sound attenuation, super-resolution acoustic imaging, and vibration mitigation.

Typical physical phenomena responsible for the band gap opening are Bragg scattering^6,7^, inherent to periodic structures, and local resonance^8^ that also promotes band gaps in materials composed of unit cells with subwavelength dimensions. The latter is the focus of the present work.

Locally resonant metamaterials operating in the nonlinear regime^9–11^ provide even more opportunities for breakthrough applications. Recently, nonlinearities have been exploited in locally resonant granular crystals^12,13^ or to obtain logic gates^14^ and acoustic diodes^15^.

Despite of great interest, the number of investigations carried out on the nonlinear dynamic behavior of locally resonant metamaterials exhibiting attenuation frequency ranges is quite limited in comparison with linear ones, also because of the conceptual and modelling difficulties^16–20^. Only few works investigated the energy transfer mechanisms induced by the nonlinear coupling between the resonator and the host medium. In some papers^21–24^, the irreversible energy transfer mechanisms are induced by a single purely nonlinear attachment, the so-called nonlinear energy sinks (NES). Only very few papers have considered cases in which nonlinear resonant attachments are densely or periodically distributed in a host material. In this case, two main energy transfer mechanisms can arise. The first one is called inter/intra-modal tunneling and is a well-studied phenomenon in nonlinear wave dynamics. It consists of an energy exchange between the wave modes of a propagating wave^25–30^. This mechanism has been shown for the first time for a discrete locally resonant chain by Lazarov and Jensen^31^ and then theoretically studied^25^ and experimental verified^26^ in elasto-acoustic metamaterials.

The second energy transfer mechanism has recently been theoretically predicted by the co-authors^32^. This new mechanism arises from a nonlinear interaction between propagating and evanescent waves triggered by autoparametric resonance, manifesting itself through the appearance of a subharmonic transmission attenuation zone^32^. This provides new, advanced means for wave mitigation and control. In Silva et al.^32^, a discrete version of a nonlinear locally resonant metamaterial with a nonlinear coupling of a neo-Hookean type, representative of rubber, between the host medium and the resonator, was studied theoretically and numerically.

In this paper, a single-material nonlinear locally resonant metastructure (i.e. dimensions of the unit cell in the order of tens of mm) is designed, and for the first time, the existence of the subharmonic attenuation zone induced by autoparametric resonance is experimentally proven. To the best of our knowledge, this represents the first experimental evidence of an emergent subharmonic band gap in a nonlinear locally resonant metamaterial.

To this aim, a locally resonant unit cell has been designed exhibiting predominantly quadratic nonlinear coupling between the host and the resonator, which, according to Silva et al.^32^, is the prerequisite for promoting the phenomenon under investigation. This has been achieved by exploiting the quadratic geometric nonlinearity arising in arched beams. Although the concept of arched resonators has been widely applied in micromechanical systems^43–48^, to the best of our knowledge, this approach has not yet been used in a practical design of metamaterials that promote the quadratic non-linearity in a local resonator. Numerical and analytical models are used to support the design process of the resonant unit cell, revealing an adequate agreement with the obtained experimental results.

## Nonlinear locally resonant metamaterial design

**Figure 1:**
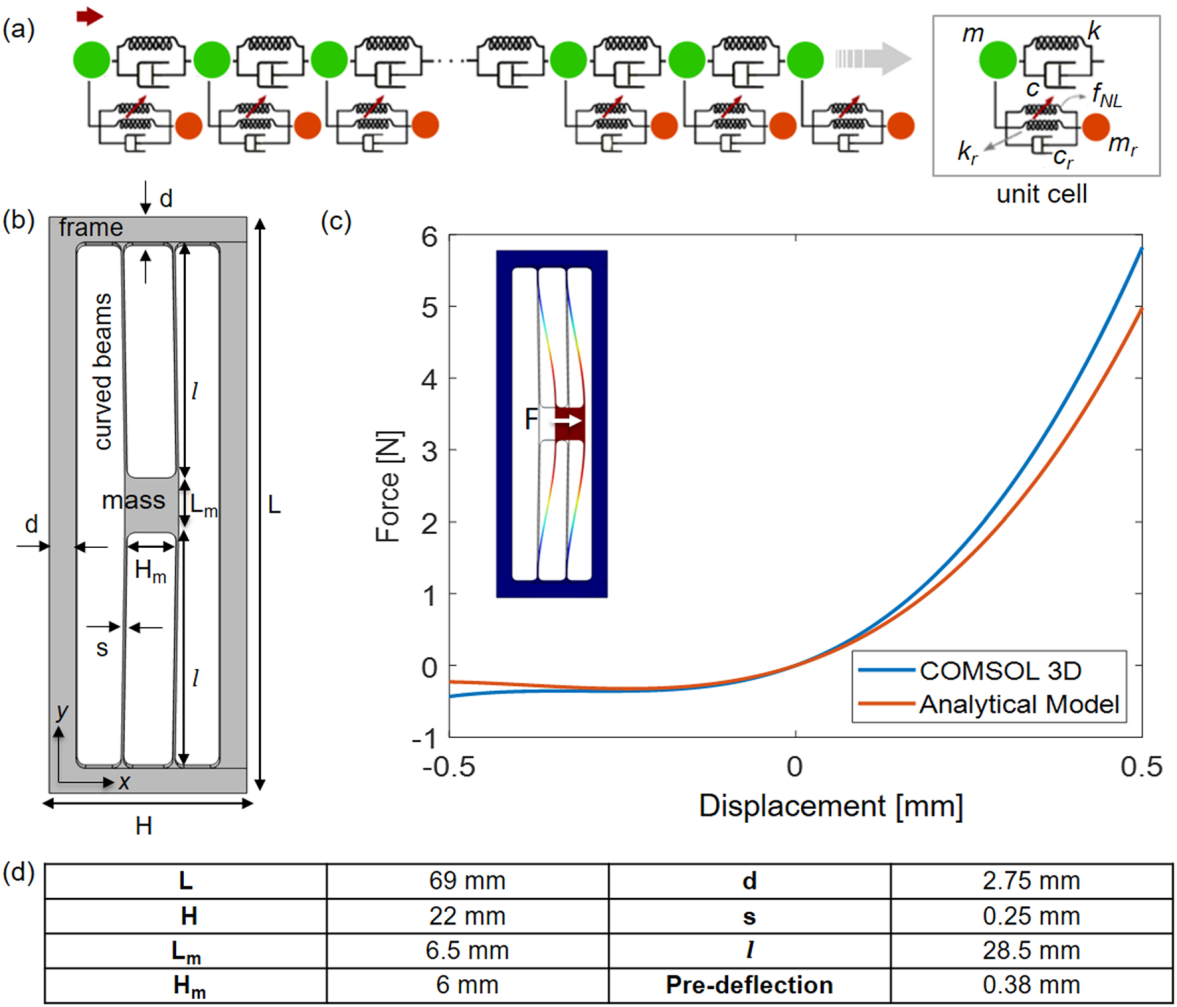
(**a**) 1D-discrete model presented in Silva et al.^32^ for a nonlinear locally resonant metamaterial, (**b**) schematic view of the proposed resonant unit cell and (**c**) nonlinear force–displacement response of the resonator computed through the analytical single degree-of-freedom model presented in the supplementary material and a 3D nonlinear static simulation in COMSOL Multiphysics. (**d**) Geometric dimensions of the unit-cell of the proposed locally resonant metastructure. The out-of-plane thickness of the unit cell (both frame and resonator) is 5 mm.

Restricting the attention to longitudinal waves, a locally resonant metamaterial can be represented by the discrete 1D model shown in Fig. 1a. The periodic main chain consists of discrete masses *m* (colored in green in Fig. 1a) that interact through linear springs of stiffness *k* and possibly dash-pots of coefficients *c*. At the same time, each mass of the main chain interacts through the other spring (*k*_*r*_) and dash-pot (*c*_*r*_) with a resonator of mass *m*_*r*_ (colored in red in Fig. 1a). These springs may exhibit a linear or nonlinear behavior and the dash-pots can be considered or neglected in the modelling process.

To exploit nonlinear phenomena, such as those leading to the appearance of a subharmonic attenuation zone, the resonator springs must be nonlinear. In particular, in Silva et al.^32^, it is shown that a quadratic nonlinearity in the relation between the force and the displacement of the resonator mass is required to promote the phenomenon. In practice, this can be achieved through different approaches, e.g. electrostatic actuation^33–35^, contact dynamics^36–38^, among others^39^. Focusing on resonators exhibiting geometric quadratic nonlinearities, several designs can be mentioned, e.g. nonlinear micromechanical cantilever system integrated with nanotubes proposed by Cho et al.^40^, the suspended cables studied by Zhao et al.^41^, the M-shape resonator designed by Leadenham et al.^42^ and the arch resonators widely proposed in the literature^43–48^, mostly not in the context of metamaterials.

### Resonant unit cell

In Fig. 1b, the geometry of the proposed resonant unit cell is shown, whereby all dimensions are reported in Fig. 1d. It consists of an external frame, corresponding to the main chain host medium, and a resonator, consisting of an inner mass connected to the frame through two arch beams. The aluminum alloy AlMgSi1 is considered as the constituent material (Young’s modulus *E* = 69 GPa and density *ρ* = 2,700 kg*/*m^3^) and no damping is taken into account.

The resonant nature of the proposed unit cell originates from the geometry of the inner resonator that is able to translate in the *x*-direction according to its first in-plane flexural mode (inset in Fig. 1c) when properly excited. The two clamped–clamped pre-deflected arch beams, which constitute the deformable part of the resonator, are the sources of the quadratic and cubic geometric nonlinear terms in its force–displacement relations. Quadratic nonlinearity arises because of the lack of the symmetry induced by the pre-deflection, while the cubic term is related to the effect of the axial load on the clamped–clamped beam for large displacements of the mass.

The design of the proposed unit cell, shown in Fig. 1b, was guided by a simplified single degree-of-freedom analytical model, presented in the supplementary material. Despite the strong simplifying hypotheses made in the analytical model (see Supplementary Information), the force–displacement response, predicted by the analytical model compares well with the results obtained from a fully 3D nonlinear static simulation performed in COMSOL Multiphysics, see Fig. 1c. The two curves show a convincing agreement; the discrepancy for very large displacements is mainly due to the 3D effects neglected in the 1D analytical model based on the beam theory and from the other hypotheses made in the analytical model. From Fig. 1c, the asymmetric nonlinear behavior of the resonator for negative and positive displacements of the mass can be clearly identified, which is required to promote the considered subharmonic autoparametric resonance energy exchange mechanism^32^.

The proposed one degree-of-freedom model is particularly useful in the design process of the nonlinear locally resonant metamaterial. By adjusting the initial shape of the arch beam through the parameter *h* (Fig. 1 of Supplementary Information), which quantifies the pre-deflection of the beams, it is possible to control the relative magnitude of the quadratic term compared to the cubic one and optimize the geometry accordingly. In particular, as shown in Silva et al.^32^, to achieve a half-subharmonic attenuation in the metamaterial under study, the quadratic contribution needs to be dominant over the cubic one.

### Linear behavior

**Figure 2:**
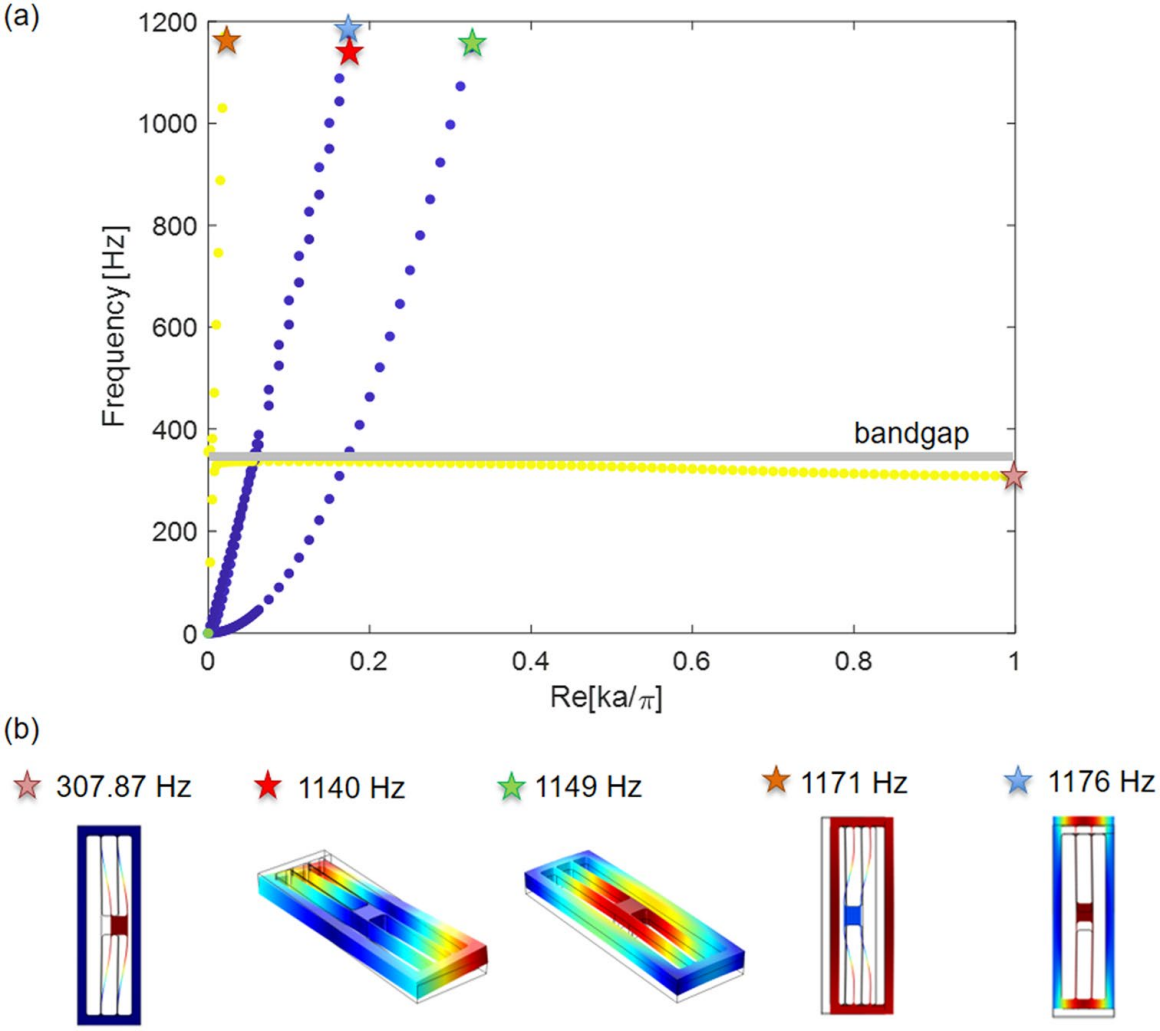
(**a**) Dispersion diagram. Only the Γ − *X* path of the Brillouin zone is considered since the interest is here restricted to longitudinal waves propagating along the chain; $\mathbf{a}$ is the dimension of the unit cell. (**b**) Modal shapes corresponding to different propagating waves. The contour of the normalized displacement field is shown in color.

Under the assumption of an infinitely periodic linear locally resonant metamaterial, the analysis of wave dispersion can be restricted to a representative unit cell by applying Bloch’s theorem^49^. For the unit cell of Fig. 1b with the dimensions reported in Fig. 1d, the natural frequency of the in-plane flexural mode computed in COMSOL Multiphysics is equal to 335 Hz. In Fig. 2, the dispersion diagram^50–54^ computed using COMSOL Multiphysics for the 3D geometry of the unit cell shown in Fig. 1b is also depicted. Dispersion curves of the propagating waves are reported in the figure together with the unit cell deformation modes corresponding to selected positions on the dispersion plot. Colors represent the wave polarization: yellow dots indicate waves polarized in the *x*-direction, while blue dots denote waves with polarization in the plane perpendicular to the *x*-axis. A band gap is visible around the natural frequency of the in-plane flexural mode of the resonator (i.e. shaded area in Fig. 2), as expected from the theory.

Next, a locally resonant metastructure made of 50 unit cells is considered, having in mind the subsequent experimental verification of the theoretical results. The number of unit cells (i.e. 50) in the chain has been chosen to compromise between three important requirements for the metastructure under study. (1) The chain must be long enough to allow the complete development of the dynamic behavior of the resonators along the chain, thus providing the expected interaction between the propagating and evanescent waves. Based on the numerical simulations, this minimum length was estimated to be at least 45 cells. (2) Manufacturability of the structure. A monolithic structure, manufactured from a single plate was preferred, to avoid introduction of internal interfaces due to joining techniques. (3) The need to exclude the presence of global modes at frequencies around approximately two times the frequency of the attenuation peak, in order to be able to numerically and experimentally detect the subharmonic attenuation peak arising from the autoparametric resonance. Finally, it should be noted that the chosen length of the metastructure is not limiting and that metastructures of different length will also exhibit the appearance of subharmonic attenuation zones, provided that the above factors are taken into consideration.

**Figure 3:**
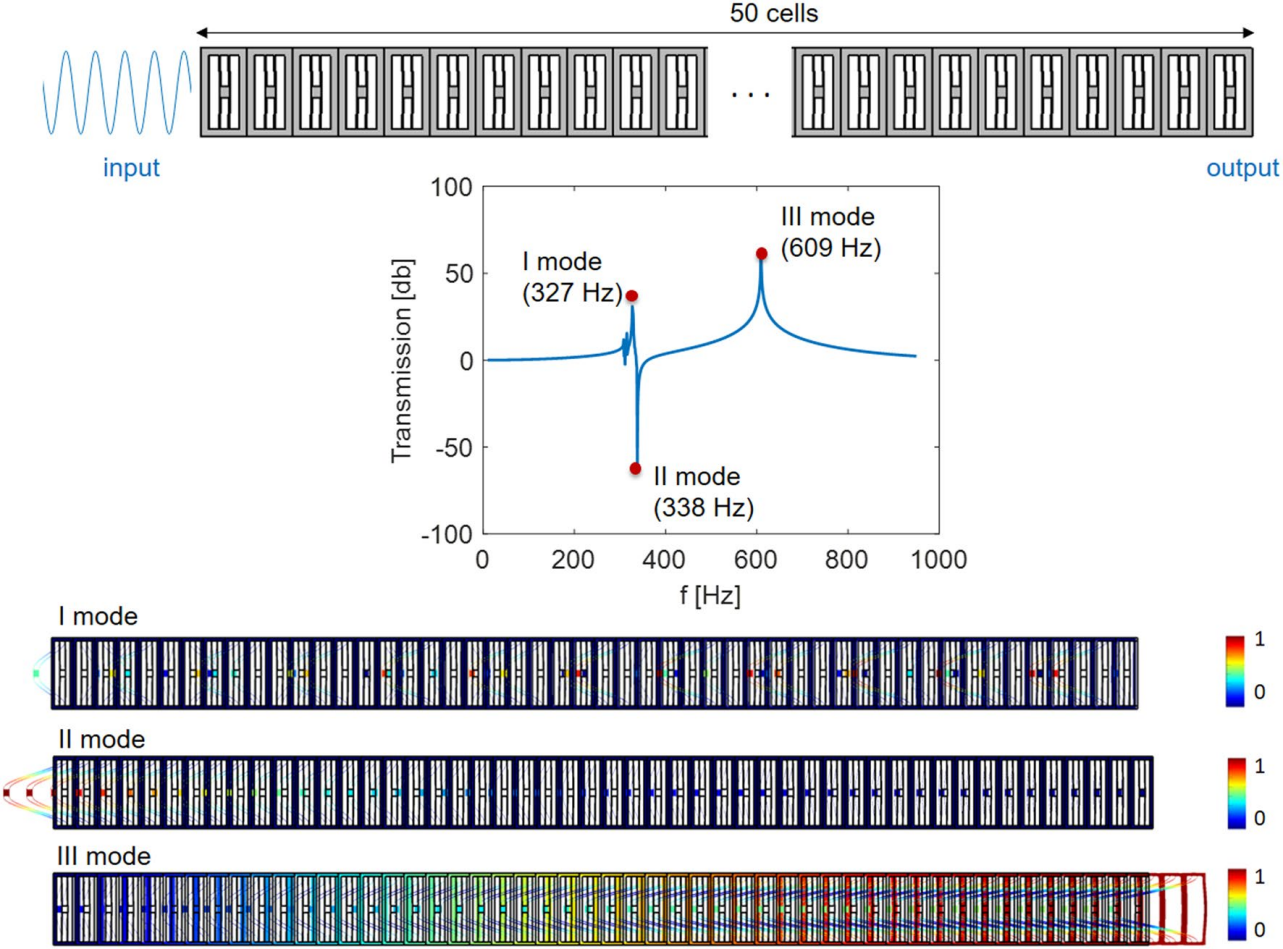
Transmission diagram computed numerically using COMSOL Multiphysics for 3D model of the chain made of 50 unit cells. The three main mode shapes for the frequency range of interest and their associated natural frequencies are also depicted. The color shows the normalized total displacement field.

The metastructure is excited at the left end with a harmonic horizontal displacement signal, while all the other edges are traction-free. The computed transmission diagram is reported in Fig. 3. The attenuation peak takes place at a frequency of 338 Hz, which almost coincides with the natural frequency of the in-plane flexural mode of the resonator inside the unit cell. As expected for a finite structure, since no damping is considered, global modes are also present. Focusing on the frequency range around the transmission dip, two global modes are present resulting in an amplification of the input signal (modes I and III in Fig. 3).

### Nonlinear behavior

The nonlinear behavior of the proposed locally resonant metastructure is investigated through nonlinear time domain simulations in COMSOL Multiphysics. A four period burst signal at 700 Hz is applied on the left side of the chain. For comparison, both linear and nonlinear time domain analyses are performed to verify the emergence of the subharmonic attenuation zone.

**Figure 4:**
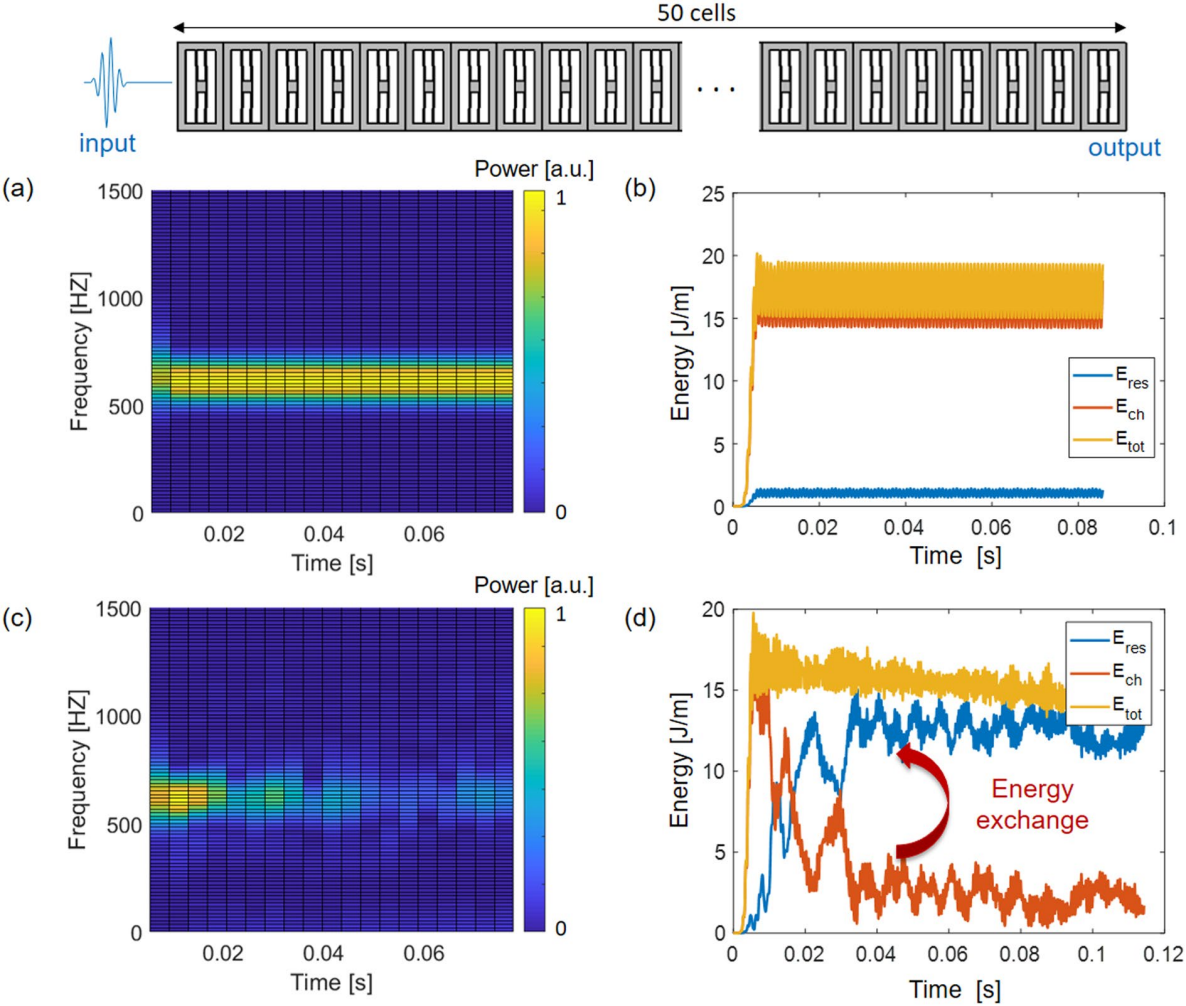
Spectrograms of the output signal computed at the right end of the chain. made of 50 cells in the (**a**) linear and (**c**) nonlinear regimes when a burst of 4 periods at around two times the frequency of the in-plane flexural mode of the resonator (i.e. 700 Hz) is applied on the left side. The spectrograms are normalized with respect to the maximum power obtained in the linear regime. The total energy (E_tot_) stored in the main chain (E_ch_) and in the resonators (E_res_) is computed for the (**b**) linear and (**d**) nonlinear cases.

In Fig. 4a, the spectrogram of the output signal computed at the right end of the chain through the time domain analysis in the linear regime is reported. As expected, the maximum transmitted power is at the input-frequency (i.e. 700 Hz) for all the time instances of the simulated interval. Figure 4b shows that the mechanical energy is stored in the main chain through which the burst signal is propagating. On the contrary, in the nonlinear regime (Fig. 4c), the spectrogram of the output signal computed at the right end of the chain differs significantly from the linear one, which is due to an energy exchange between the main chain and the resonators. This is obvious from Fig. 4d which reveals that the energy originally stored in the main chain significantly decreases after about 20 ms, when the energy transfers from the main chain to the resonators.

As explained in Silva et al.^32^, the harmonic excitation at one end of the metamaterial induces wave propagation with the frequency of the harmonic excitation, but also the near-field (evanescent) waves. For locally resonant metamaterials, the resonance of the local resonator opens up a band gap and an associated evanescent wave. In Silva et al.^32^, it was demonstrated that when the local resonance frequency is around half the excitation frequency and a quadratic nonlinear interaction exists between the host and the local resonator, the propagating wave and the half-subharmonic evanescent wave couple strongly with each other for high amplitudes of excitation, leading to parametric excitation of the evanescent wave, and energy transfer from the propagating to the evanescent wave in a similar fashion as it occurs for autoparametric resonance in nonlinear dynamical systems. For the metastructure under concern, energy that was initially imputed as a propagative wave with frequency of 700 Hz is converted to the evanescent wave with a frequency around the half-subharmonic frequency of 350 Hz, which is within the local resonance band gap. As a result, local resonators of the first unit cells are excited, reflecting back the elasto-acoustic wave, and preventing it from propagating through the chain. In other words, the energy flow to the opposite (right) end of the metastructure is significantly reduced, explaining the reduced output signal in the spectrogram plots (Fig. 4c).

### Experimental results

**Figure 5:**
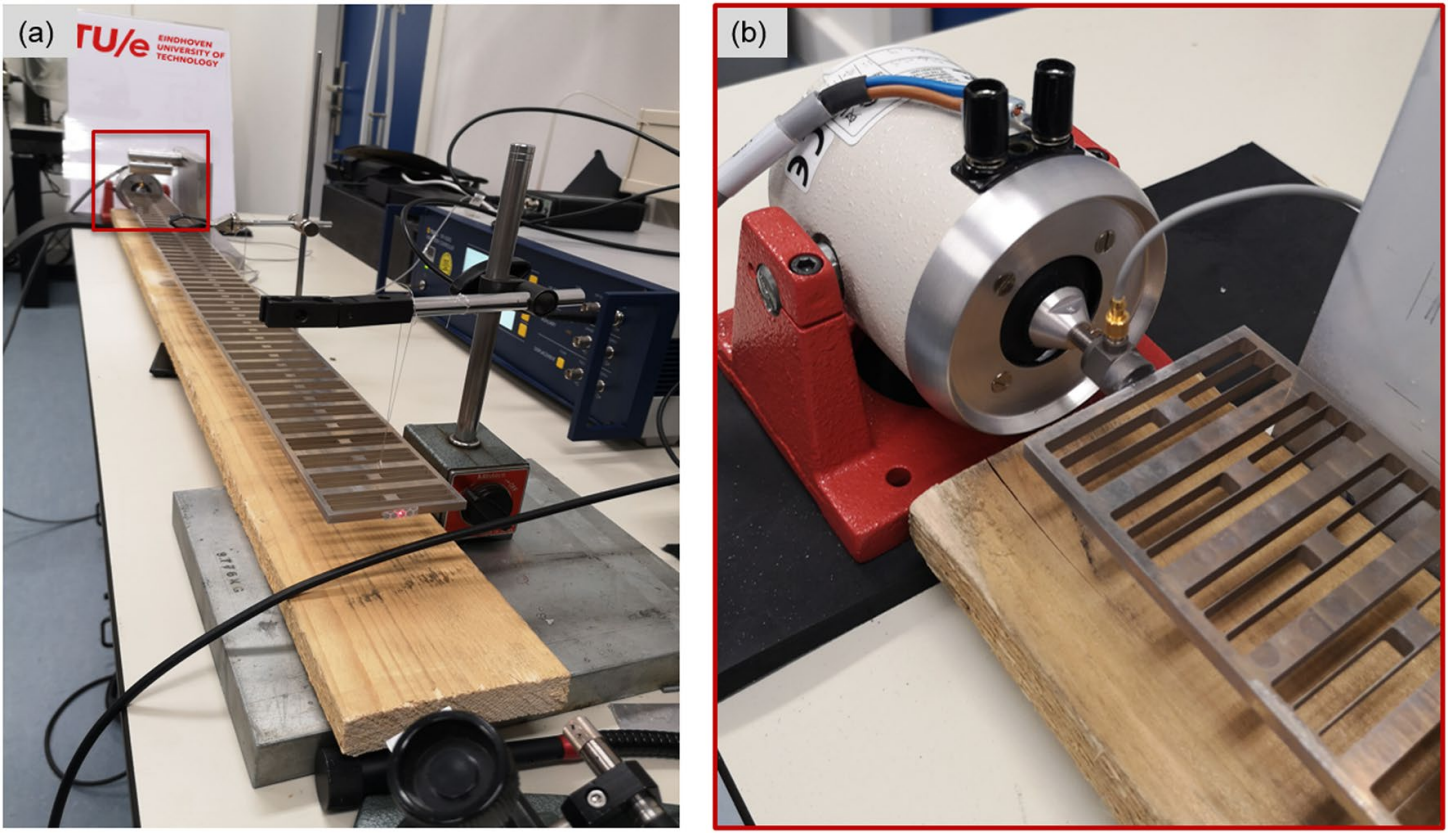
(**a**) Experimental set-up, (**b**) close-up view of the fabricated structure attached to the shaker on the left side.

The designed nonlinear locally resonant metastructure is fabricated and tested both in the linear and nonlinear regimes (by varying the amplitude of the excitation). The experimental setup is shown in Fig. 5a. During the tests, the input signal is applied to the left side of the chain through a shaker glued to the metastructure in the form of a prescribed acceleration (Fig. 5b), while the output signal is measured by the laser vibrometer as the displacement of one point on the right side of the chain.

### Linear behavior

**Figure 6:**
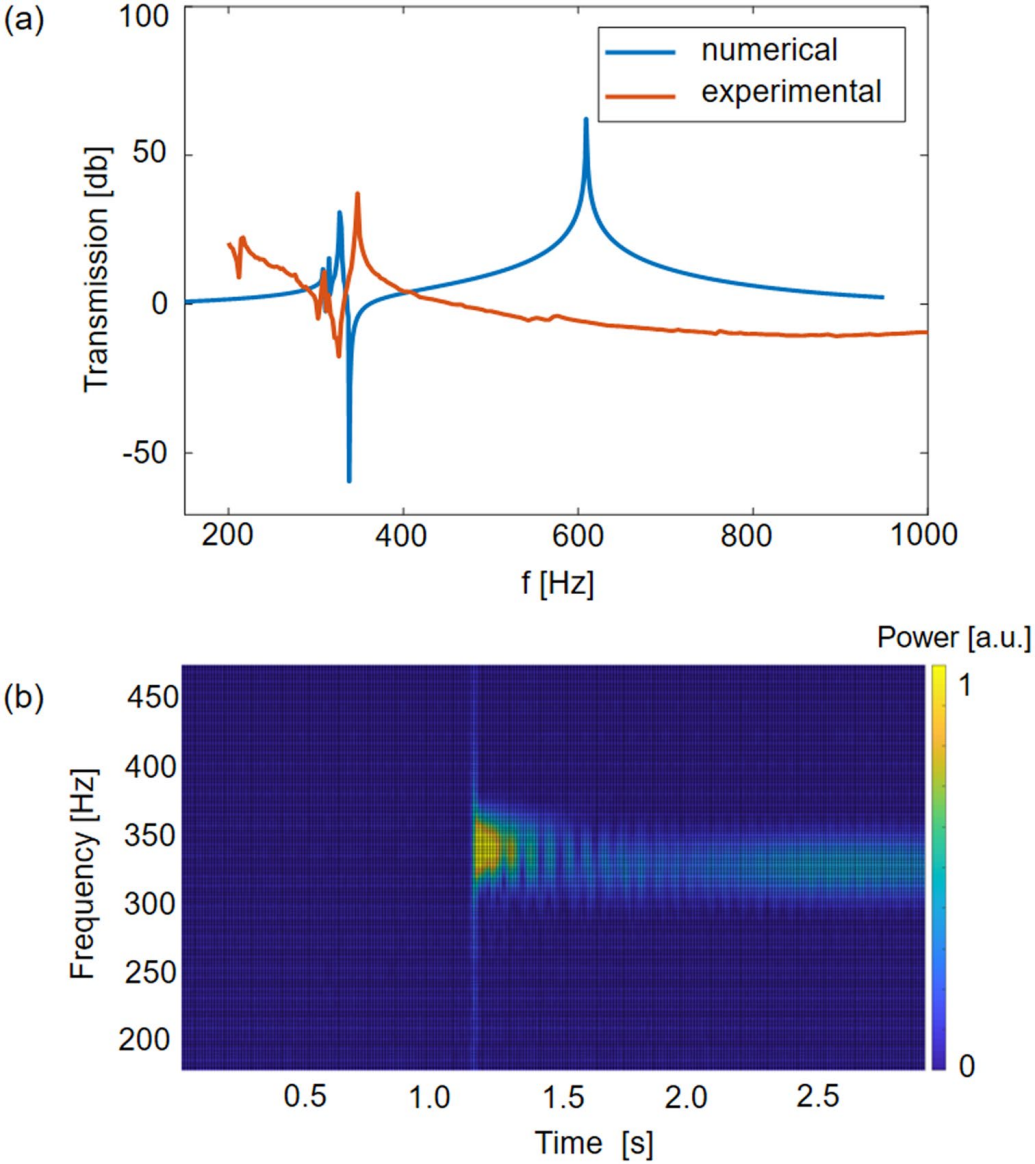
(**a**) Comparison between the numerical and experimental transmission diagrams. The frequency mismatch between the two attenuation peaks is in the order of 10 Hz, which can be attributed to fabrication imperfections and material property uncertainties. (**b**) Spectrogram of the output signal measured at the right end of the chain when a sinusoidal input signal of 0.1 V at 325 Hz is applied on the left side. The spectrogram is normalized with respect to the maximum power of the input signal (i.e. left end).

First, characterization of the metastructure in the linear regime is performed. In Fig. 6a, the experimental transmission diagram obtained by applying a frequency sweep from 200 to 1,000 Hz to the structure is compared with the numerically computed transmission. Both, the experimental and numerical results, show the clear local resonance transmission dip at around 330 Hz. The frequency mismatch between the numerical and experimental attenuation peaks of approximately 10 Hz (3% of error) can be attributed to the imperfections and material property uncertainties. The different positions of the global modes, producing peaks in the transmission diagram, can be rationalized by considering some details of the experimental set-up. The metastructure is in fact glued to the accelerometer and then to the shaker, which results in an increase of the mass involved in the global modes and hence a shift of their natural frequencies towards lower frequencies. Manual suspension of the structure, which may provide an extra restriction in the longitudinal direction, as well as the fabrication imperfections, can also mildly affect and shift the global modes of the metastructure.

Figure 6b shows the spectrogram of the output signal measured by the laser vibrometer when an input sinusoidal signal of amplitude 0.1 V (~ 25 µm, small displacements leading to a linear response) at 325 Hz is applied to the metastructure. The structure is indeed able to attenuate the signal at 325 Hz, as expected from the transmission diagram reported in Fig. 6a and from the simulations reported in the previous sections.

### Nonlinear behavior

**Figure 7:**
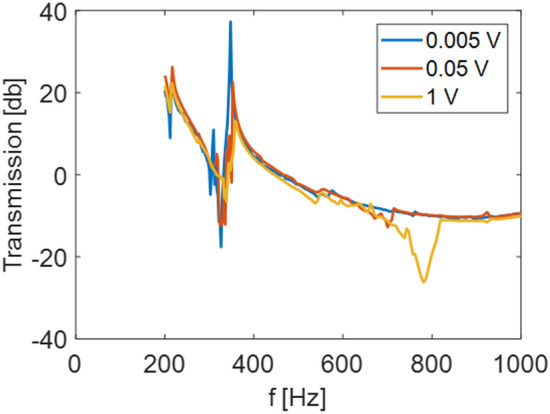
Transmission diagram measured for different amplitudes of the input signals. By increasing the amplitude of the input, the nonlinear regime is triggered and the subharmonic attenuation zone emerges.

Next, the nonlinear behavior of the proposed locally resonant metastructure is studied. In Fig. 7, the experimental frequency sweep transmission diagrams for increasing amplitudes of the input signal are reported. As expected from the theoretical study presented in Silva et al.^32^ and from the numerical simulations reported here, the appearance of the subharmonic attenuation zone is more pronounced when the amplitude of the input signal increases, whereby the nonlinearity becomes more pronounced. This provides an experimental proof of the fact that (i) the level of attenuation around the natural frequency of the resonator slightly decreases with the increase of the input amplitude, and (ii) a second attenuation zone emerges around two times the natural frequency of the resonator for higher excitation amplitudes. The first observation is strictly related to the intrinsic amplitude-dependent response of nonlinear systems, periodic or not, while the second one relates to the discovered phenomenon of subharmonic attenuation zone induced by autoparametric resonance in locally resonant metamaterials^32^.

**Figure 8:**
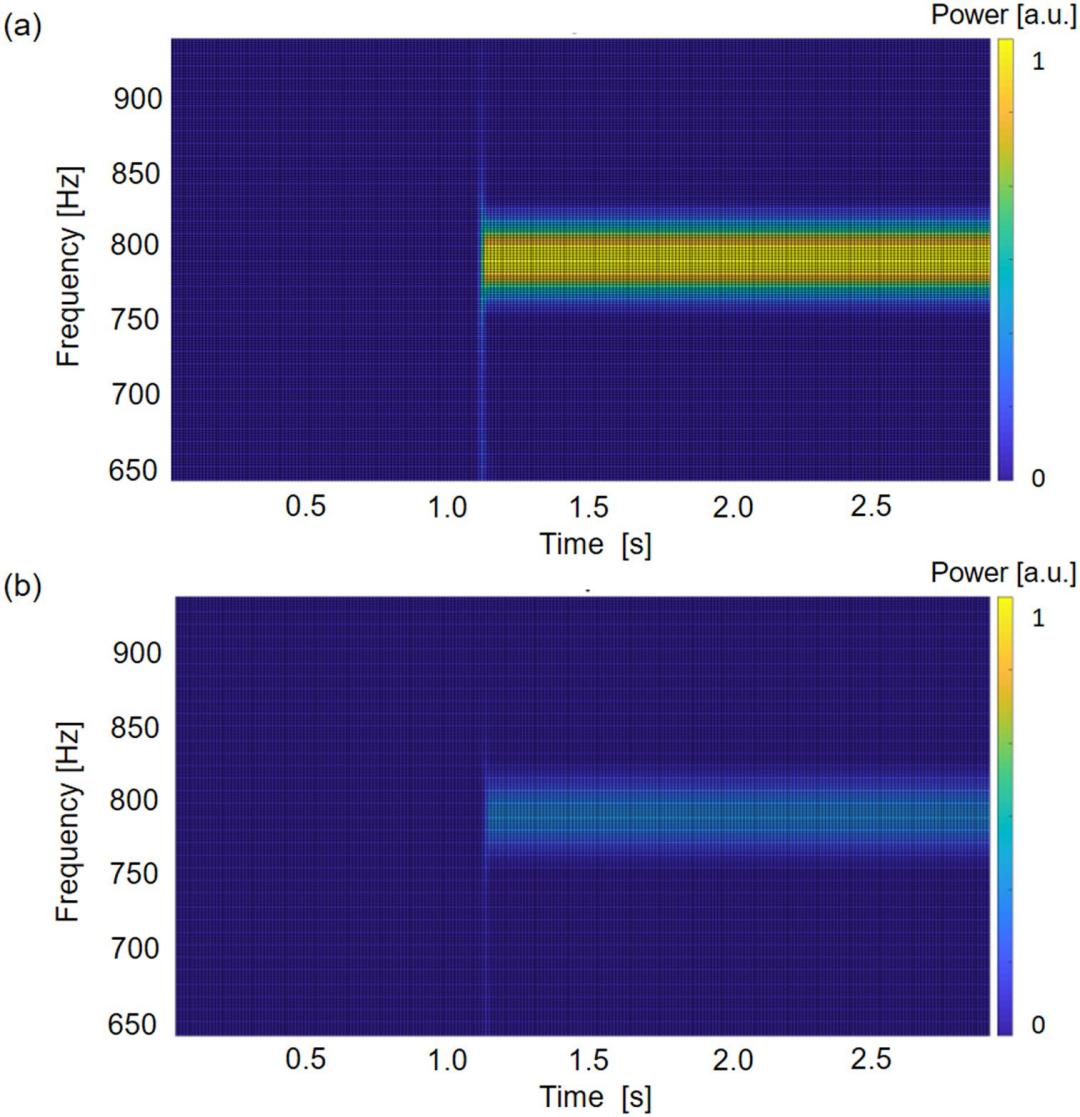
Spectrogram of the signal measured at the (**a**) left and (**b**) right ends of the metamaterial chain when a sinusoidal input at 790 Hz is applied to the structure. The spectrograms are normalized with respect to the maximum power of the input signal (i.e. left end).

To further investigate the experimental evidence of the subharmonic attenuation zone induced by autoparametric resonance, the time dependent response of the metastructure for an input signal of amplitude 1 V (~ 45 µm, large displacements) at 790 Hz is addressed. In Fig. 8, the spectrograms of the input and output signals are reported, which convincingly shows that the metastructure indeed attenuates the excitation at this frequency.

## Discussion

The presented results clearly demonstrate the energy exchange between the interacting primary and subharmonic wave modes due to autoparametric resonance. To the authors’ best knowledge, this is the first evidence providing experimental validation of this phenomenon in a single-material locally resonant metamaterial.

A unit cell consisting of a host frame and an inner nonlinear resonator is properly designed, yielding a locally resonant metastructure that exhibits a subharmonic attenuation induced by autoparametric resonance. A prototype metastructure has been fabricated and experiments show a convincing agreement with theoretical predictions.

These results lay the basis for the emergence of a new generation of high-performance nonlinear locally resonant metamaterials. This opens new perspectives in the modelling and fabrication of tunable metamaterials with amplitude-dependent attenuation zones occurring at harmonics multiple of the local resonance frequency, ultimately leading to novel applications, such as: tunable and multi-harmonic filters and imaging devices.

## Methods

### Dispersion diagram

According to the Bloch-Floquet expansion theorem^49^, the displacement field **u** can be expressed as:

1 $${\varvec{u}}\left(\mathbf{x},\mathbf{k},t\right)={\varvec{U}}{e}^{i\left(\mathbf{k}\bullet \mathbf{x}-\omega t\right)}$$

where **x** is the position vector, **k** the wave vector, ω the frequency and **U** a periodic Bloch displacement vector. In this work only the Γ*-X* path of the Brillouin zone is considered since the focus here is only on the longitudinal waves propagating along the chain of the resonant unit cells.

To obtain the dispersion diagram for the unit cell shown in Fig. 1b, the commercial software COMSOL Multiphysics is used: Bloch-Floquet periodic boundary conditions are applied along the *x*-direction on the left and right sides of the 3D unit cell while all the other surfaces are left free.

### Linear regime transmission diagram

The transmission diagram in the linear regime is computed in COMSOL Multiphysics through a frequency-domain analysis. A sinusoidal input signal of amplitude 10 µm is applied on the left side of the chain in the form of a prescribed displacement in the *x*-direction and the output displacement is computed as the displacement averaged along the height of the right side of the chain as schematically shown in the top part of Fig. 3. The right side of the chain is left free in correspondence with the experimental setup. The 3D geometry of the chain is discretized with quadratic finite elements. The frequency of the input signal is swept from 1 to 950 Hz with steps of 1 Hz. No damping is considered in this simulation according to the hypothesis on the lossless material made throughout this work.

### Nonlinear time domain analysis

A nonlinear time domain analysis is performed to simulate the propagation of the elastic wave through the chain. For the numerical analyses in the nonlinear regime, a four-period burst signal of amplitude 50 µm is applied at the left side of the chain in the form of a prescribed horizontal displacement (along the *x*-direction). The right side of the chain is left free in correspondence with the experimental setup.

Thanks to the geometric dimensions of the metastructure, i.e. a small thickness compared to its in-plane dimensions, 2D analyses under a plane-stress assumption are performed, significantly reducing the computational effort compared to full 3D simulations. Quadrilateral quadratic Lagrange finite elements are used for the discretization of the unit cell geometry, with the size of the elements small enough to resolve the propagating elastic wave in space. An implicit generalized-*α* method is used for the time integration, with a time step small enough (λ_min_/30) to resolve all the frequencies actuated by the transient burst signal.

### Experimental set-up

The metastructure consisting of 50 unit cells was fabricated by a standard electrical discharge machining (EDM) process from a monolithic AlMgSi1 sheet of 5 mm thickness.

The fabricated chain is suspended through fishing wires as shown in Fig. 5a to simulate the free condition at both top and bottom surfaces of the metastructure. The total experimental set-up employed for the measurements is described in the Supplementary Information.

## Supplementary information

Supplementary information.
