# Supplementary material for: Experimental proof of emergent subharmonic attenuation zones in a nonlinear locally resonant metamaterial
Source: Sci Rep. 2020 Jul 21;10:12041. doi: 10.1038/s41598-020-68894-3 (PMC7374692; doi:10.1038/s41598-020-68894-3)
Supplement: Supplementary file 1 — Supplementary information. [file 41598_2020_68894_MOESM1_ESM.docx]

**Experimental proof of emergent subharmonic attenuation zones in a nonlinear locally resonant metamaterial**

Valentina Zega^1*^, Priscilla B. Silva^1^, Marc G. D. Geers^1^, Varvara G. [Kouznetsova](https://www.scopus.com/authid/detail.uri?origin=AuthorProfile&authorId=6603501386&zone=)^1^

^1^ Eindhoven University of Technology P.O. Box 513, 5600 MB Eindhoven, The Netherlands

*Corresponding Author:

zega.valentina@gmail.com

+390223996213

**Supplementary Information**

1. **Analytical dynamic model for the arch beams**

A clamped-clamped arch beam of length l, in-plane thickness s and out-of-plane thickness t is shown in Fig. 1.


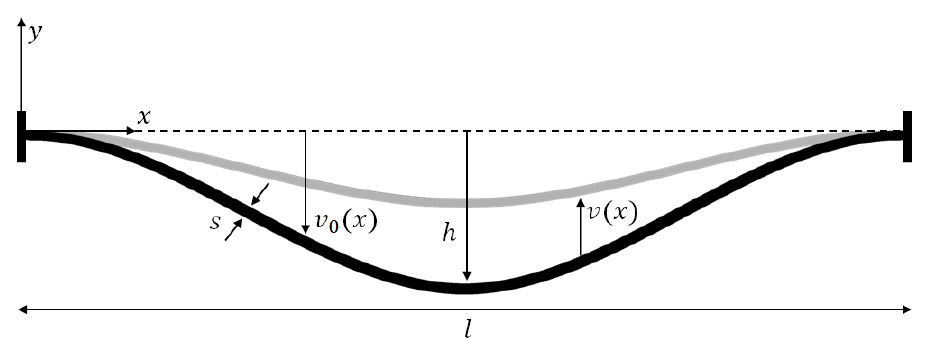


Figure 1: Schematic view of an arch beam.

Denoting by E the effective Young’s modulus of the material, ρ its density, $I=1/12s^{3}t$ the moment of inertia and A the area of the beam cross-section, it is possible to write the governing equation of motion based on the Euler-Bernoulli beam assumptions^1,2^ as:

$EIv^{IV}-\frac{EA}{2l}\int_{0}^{l} \left( v^{'2}+2v^{'}v_{0}^{'} \right)dx\left( v^{''}+v_{0}^{''} \right)+\rho A\ddot{v}=F,$ (1)
where $v\left( x,t \right)$ describes the deflection of the beam in the positive *y*-direction, $v_{0}\left( x \right)$ represents the initial shape of the arch and *F*(*t*) is an external load. Note that $\dot{(\cdot)}$ and $(\cdot)'$ represent the derivatives with respect to time and space, respectively. Moreover, in eq. (1) it is assumed that $s \ll l$, $h \ll l$ and the deflections are small with respect to the beam length.

Boundary conditions at the fixed ends of the arch, read:

$v\left( 0,t \right)=v\left( l,t \right)=0, \frac{\partial v}{\partial x}\left( 0,t \right)=\frac{\partial v}{\partial x}\left( l,t \right)=0.$ (2)
A numerical solution for eq. (1) can be obtained expressing $v\left( x,t \right)$ in terms of a set of generalized coordinates *V*(*t*) multiplied by known space functions Φ(*x*). In the spirit of the Rayleigh method a single generalized variable *V*(*t*) representing the maximum displacement of the beam in time, can be introduced and the approximate solution for the arch oscillation can be searched in the form:

$v\left( x,t \right)=V\left( t \right)\Phi\left( x \right);$ (3)
Φ(*x*) = cosh(*λ*_1_*x*) *-* cos(*λ*_1_*x*) + *β*_1_(sinh(*λ*_1_*x*) *-* sin(*λ*_1_*x*)) being the first eigenfunction of the clamped- clamped straight beam with *λ*_1_ = 4*.*73 and *β*_1_ = (cos(*λ*_1_) *-* cosh(*λ*_1_))*/*(sinh(*λ*_1_) *-* sin(*λ*_1_)). In the following it is assumed that the initial shape of the arch beam is described by $v_{0}(x)$ = *h*Φ(*x*) for the sake of simplicity.

Once the approximate solution (3) is substituted in the equation of motion, the weak form is obtained by multiplying (1) with the test function Φ(*x*) and integrating in space. The equation of motion reads:

$EIV\left( t \right)\int_{0}^{l} \left( \Phi\left( x \right)^{''} \right)^{2}dx+\frac{EA}{2l}\left( V\left( t \right)^{3}\left( \int_{0}^{l} \left( \Phi\left( x \right)^{'} \right)^{2}dx \right)^{2}+3hV\left( t \right)^{2}\left( \int_{0}^{l} \left( \Phi\left( x \right)^{'} \right)^{2}dx \right)^{2}+ \right.$

$\left. + 2V\left( t \right)h^{2}\left( \int_{0}^{l} \left( \Phi\left( x \right)^{'} \right)^{2}dx \right)^{2} \right)+\rho A\ddot{V}\left( t \right)\int_{0}^{l} \Phi\left( x \right)^{2}dx= \int_{0}^{l} F\Phi\left( x \right)dx.$ (4)

Equation (4) can be re-written in the more compact form

$m\ddot{V}\left( t \right)+k_{1}V\left( t \right)+k_{2}V\left( t \right)^{2}+k_{3}V\left( t \right)^{3}= \bar{F}$ (5)
where
$m=\rho A\int_{0}^{l} \Phi\left( x \right)dx,$ (6)
$k_{1}=EI\int_{0}^{l} \left( \Phi\left( x \right)^{''} \right)^{2}dx+\frac{EA}{l}h^{2}\left( \int_{0}^{l} \left( \Phi\left( x \right)^{'} \right)^{2}dx \right)^{2},$ (7)
$k_{2}=3h\frac{EA}{2l}\left( \int_{0}^{l} \left( \Phi\left( x \right)^{'} \right)^{2}dx \right)^{2},$ (8)
$k_{3}=\frac{EA}{2l}\left( \int_{0}^{l} \left( \Phi\left( x \right)^{'} \right)^{2}dx \right)^{2},$ (9)
$\bar{F}=\int_{0}^{l} F\Phi(x)dx$ (10)
Equation (5) describes the nonlinear dynamics of the arch beam under the one degree-of-freedom approximation (3). As expected, if the beam is initially straight (i.e. *h* = 0), the symmetry is recovered and the quadratic term disappears. On the other side, the cubic term in (5) does not depend on the initial shape of the beam, being related to the axial force that arises in the clamped-clamped beam when the large displacement regime is entered.

1. **Analytical dynamic single degree-of-freedom model for the resonator**

The analytical dynamic single degree-of-freedom model for the resonator located inside the unit cell of the proposed metastructure is obtained starting from the results of the previous section and using further simplifying hypotheses.

As shown in Fig. 1a of the paper, the single degree-of-freedom model of the resonator is fully described by an equivalent mass (*m*_r_ in Fig. 1a of the paper) and equivalent linear (*k*_r_) and non-linear (*f*_NL_) stiffnesses (see Fig. 1a of the paper). The equivalent point mass is then identified with the mass of in-plane dimensions *L*_m_ x *H*_m_, while the equivalent stiffnesses correspond with the stiffness of the arch beams connecting the mass to the frame.

The equivalent stiffness is here computed considering two arch clamped-clamped beams connected in parallel. The length of each beam in the analytical model is 2*l + L_m_* = 63.5 mm, i.e. the lengths of the two beams in the unit cell plus the length of the central mass. This is a simplifying hypothesis since in a standard clamped-clamped beam only one point (i.e. the mid-point) experiences the maximum deflection, while in the actual unit cell the mass enforces a constant displacement of a part of the beam of length *L*_m_. Once the stiffnesses $k_{i}, i=1,2,3$ of one clamped-clamped arch beam are computed through the model reported in the previous section by putting *l* = 63.5 mm, the equivalent stiffnesses are computed by considering the two arch beams in parallel as $K_{i} = 2k_{i}$ by considering the two arch beams in parallel.

The equation of motion for a resonator in 1D model then reads:

$M\ddot{V}\left( t \right)+K_{1}V\left( t \right)+K_{2}V\left( t \right)^{2}+K_{3}V\left( t \right)^{3}= \bar{F}$ (11)

where

*M* = 2*m* + *m_r_*_,_ (12)

*K*_1_ = 2*k*_1_ (13)

*K*_2_ = 2*k*_2_ (14)

*K*_3_ = 2*k*_3_ (15)

1. **Experimental set-up**

A computer connected to a Data Acquisition System DSP SIGLAB 20-42 controls the amplitude and frequency content (e.g. sinusoidal input or frequency sweep) of the input signal, which is then amplified through a Bruel & Kjaer Amplifier LDS LPA 100 and transmitted to a Bruel & Kjaer V201 shaker. The shaker oscillates according to the received input signal, thus providing the excitation to the locally resonant metastructure. The elastic waves excited in the metastructure propagate (or attenuate depending on the frequency content) and the output signal is measured on the opposite side of the chain by a vibrometer Polytec OFV-552 interfaced with a controller Polytec OFV-5000 and then sent to the Data Acquisition system directly connected with the computer. To measure the acceleration at the input, an accelerometer Bruel & Kjaer 31993, type 4533-B is attached to the shaker on its left side and glued to the metastructure on its right-hand side. The accelerometer sends the signal through the Bruel & Kjaer 1704 conditioner back to the Data Acquisition System.


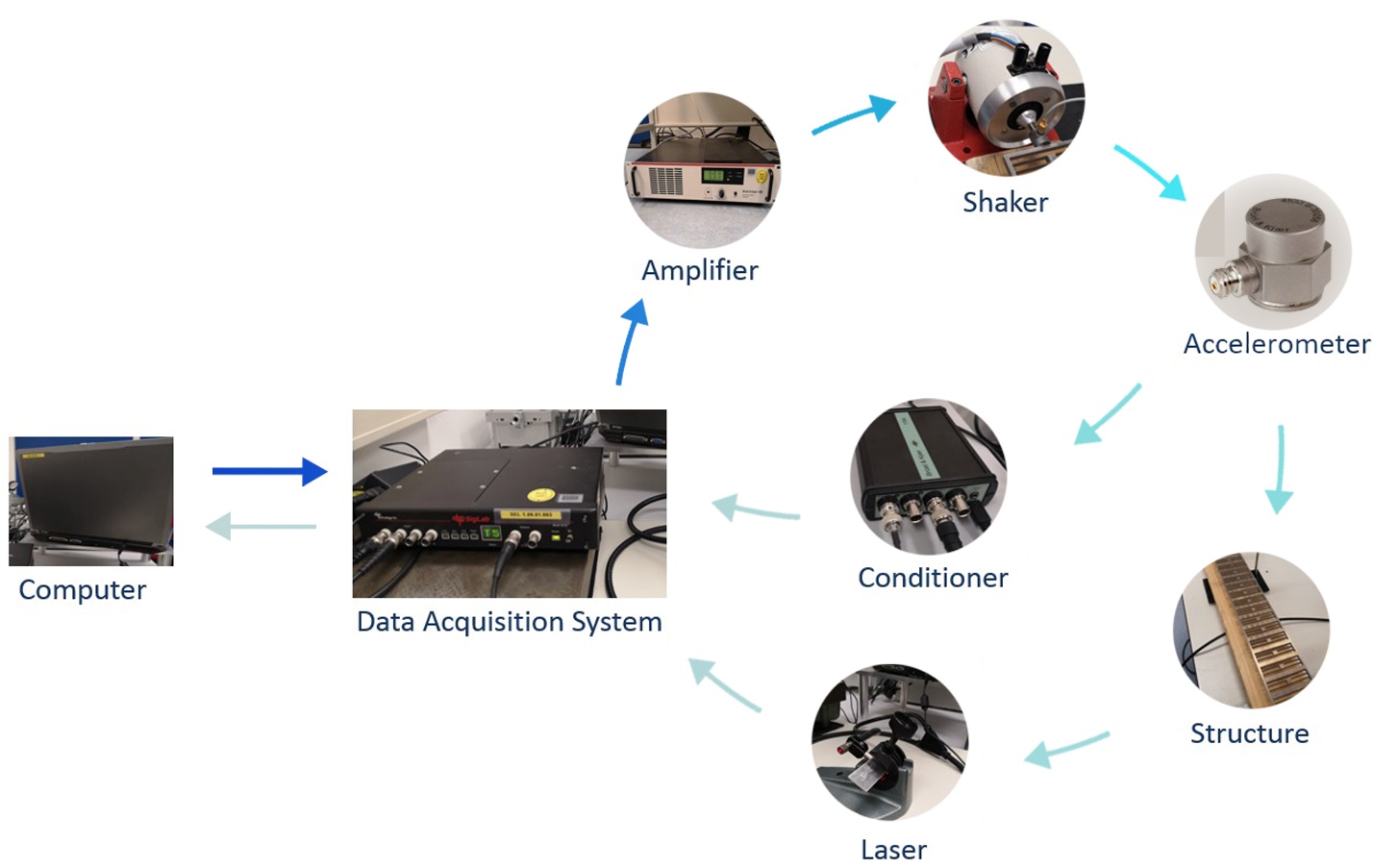


Figure 2: Experimental set-up employed for the measurements.

1. **Numerical simulations and experimental tests: a comparison**

In Table 1, the main differences between numerical simulations and experimental tests are summarized in order to provide a clear framework for the interpretation of the results shown in the paper.

Table 1: Comparison between numerical simulations and experiments

|  | **Numerical Simulation** | **Experiments** |
| --- | --- | --- |
| Input | Applied as a prescribed displacement on the left side of the first cell of the metastructure. | Applied as a prescribed acceleration through a shaker glued to an accelerometer, which is itself glued to the left side of the first cell of the metastructure. |
| Output | Computed as the displacement averaged along the height of the right side of the last cell of the metastructure. | Measured by the laser vibrometer as the displacement of one point on the right side of the last cell of the metastructure. |

**References**

1. Leadenham S et al M-shaped asymmetric nonlinear oscillator for broadband vibration energy harvesting: Harmonic balance analysis and experimental validation *J. of Sound and Vibr.* **333** 62096223 (2014)
2. Hajjaj A Z et al The static and dynamic behavior of MEMS arch resonators near veering and the impact of initial shapes *Int. J. Nonlin. Mech.* **95** 277-286 (2017)
